# Evaluating fishermen’s conservation attitudes and local ecological knowledge of the European sardine (*Sardina pilchardus*), Peniche, Portugal

**DOI:** 10.1186/s13002-017-0154-y

**Published:** 2017-05-05

**Authors:** Heitor O. Braga, Ulisses M. Azeiteiro, Henrique M. F. Oliveira, Miguel A. Pardal

**Affiliations:** 1 Centre for Functional Ecology - CFE, Department of Life Sciences, University of Coimbra, Calçada Martins de Freitas, 3000-456 Coimbra, Portugal; 2 CAPES Foundation, Ministry of Education of Brazil, Caixa Postal 250, 70040-020 Brasília, DF Brazil; 3 Department of Biology & CESAM - Centre for Environmental and Marine Studies, University of Aveiro, 3810-19 Aveiro, Portugal; 4 Environmental analyst at Health, Safety & Environment, 04552-000 Vila Olímpia, São Paulo, SP Brazil

**Keywords:** Attitudes, Local ecological knowledge, European sardine, Fishermen, Conservation, Portugal

## Abstract

**Background:**

European sardines are an important fishing resource in the North Atlantic. Recognized for its great commercial and economic value in southern Europe, this resource currently has low stock indices. From this perspective, fishers’ local ecological knowledge (LEK) is appreciated as an auxiliary tool in the management of sardines in this region. Our goal is to evaluate the LEK and attitudes towards the conservation of *Sardina pilchardus* in the typical fishing village of Peniche, Portugal.

**Methods:**

From June to September 2016, we carried out 87 semi-structured interviews. The four main points of the interviews were interviewee profile, fishing structure, fishermen’s LEK and attitudes towards sardine conservation. The interviews were qualitatively and quantitatively analyzed using a 3-point Likert scale. An LEK index and an attitude index were generated. Comparison analyses and correlations were made between the indices and variables of the interviewee profile and the fishing structure.

**Results:**

The mean LEK index was 0.55 and was classified as moderate. The attitudes index in relation to conservation was 0.76 and was classified as positive. This index had a positive and significant correlation with the LEK index and a significant negative correlation with the fishermen’s age. When the LEK index was compared with the educational level, significant differences were observed only between class A and class C. The result showed that the differences in the attitudes index were statistically significant when the three educational classes were compared.

**Conclusions:**

The fishermen of Peniche in Portugal present moderate informal knowledge about the biology and ecology of sardines. Attitudes towards conservation were predominantly positive. Fishermen with greater LEK, with a higher educational level and at a younger age presented more positive attitudes in relation to environmental conservation issues in the present case of the sardine population. The LEK is not necessarily related to the educational level of the fishermen. We suggest environmental education programs for the communities that depend on this resource. The use of LEK and fishermen’s perceptions can help in the management of the European sardine fishery in Portugal.

**Electronic supplementary material:**

The online version of this article (doi:10.1186/s13002-017-0154-y) contains supplementary material, which is available to authorized users.

## Background

The European sardine (*Sardina pilchardus*, Walbaum, 1792) is a small pelagic fish species from the family Clupeidae that is widely distributed in the northeast Atlantic Ocean, from Iceland and the North Sea to Mauritania and Senegal, and in the Mediterranean Sea, Sea of Marmara and Black Sea [1]. This species is a schooling and oceanodromous fish that occurs in several environments in the world’s oceans [1], mainly at depths between 10 and 100 m (often above 25 m) [2, 3]. It is a warmer-water fish [4], and it is usually found between the coastline and up to 100 km offshore [3].

The European sardine is a relatively short-lived species that exhibits rapid growth and high fertilization capacity [5]. The main components of the sardine’s diet are zooplankton and phytoplankton [6, 7]. Sardines are an important food item for marine mammals, predominantly the common dolphin (*Delphinus delphis,* Linnaeus, 1758), as well as seabirds and other, larger fish species [6, 8, 9]. Sardines allocate energy resources to grow predominantly during spring and summer [10]. The spawning season occurs over a broad spectrum between October and April [11] and is more pronounced in the months of December and February [10].

Sardines support an important fishery in Atlantic Iberian waters [12]. This important species of the North Atlantic is captured through purse-seine fisheries by Portuguese vessels [13]. It is recognized for its high commercial value and is responsible for a large part of the volume of fish discharged in these coastal waters [13–15]. In Portugal, the sardine has great socioeconomic importance and is associated with recognized traditions for the Portuguese population [15]. This fishing resource can be used for fresh food, bait for other fisheries and the canning industry [16, 17].

The northwestern part of Portugal is the main area of recruitment for this pelagic species [13]. However, the level of recruitment in recent years has not been sufficient to recover the abundance of the species in coastal areas [14]. In 2012, 38% of the catches of sardines in European waters were carried out by Portugal, indicating a decrease compared to the previous year [13]. Regarding stock development over time, despite a decrease in observed fishing pressure in 2016, the recruitment of the species has been below the long-term average since 2005, and the biomass of fish aged 1 and older is at a low level [18]. Although fishing limits imposed since 2011 have contributed to the reduction of fishing pressure, stocks remain at very low levels [18].

Effects of climate variability may also lead to changes in the sardine populations [4]. As a pelagic species, the sardine plays a prominent intermediary ecological role within the marine ecosystem [19]. Moreover, with other pelagic species, it may contribute to the modification of the structure and functioning of the marine ecosystem as a result of eutrophication, overfishing and climate change [20, 21]. Due to all these factors, this pelagic fish, which represents the most abundant catch of the Portuguese fleet, is an important but difficult challenge for defining management goals [10].

The current international policy of environmental resources management requires a change of paradigms through the inclusion of more delineated human perspectives on the environment [22]. In practice, the applicability of the knowledge of local actors about changes in the ecosystem being managed is based on processes that generate their knowledge, experience and concept of the dynamics of the environment [23]. The combination of this institutional and organizational background with the ecological scale can make co-management more robust against external environmental adversities [24]. Thus, socio-ecological approaches must be integrated into a wide spectrum and actively conducted and strengthened to improve a more participatory and adaptive governance of coastal systems [25].

Ethnozoology science studies are increasingly used in zoological and ecological research programs [26]. Specifically ethnoichthyological studies have been increasingly highlighted in recent years as an auxiliary tool in the management of fishery resources [27–30]. In this perspective, local ecological knowledge (LEK), meaning the specific knowledge of an individual about the environment [31], can be used as a complementary way to conduct a more equitable and culturally sensitive approach to new conservation programs [32]. Additionally, it becomes useful to understand people’s attitudes towards a particular resource to determine the effectiveness of management programs by showing their successes and failures [33]. Therefore, the main goal of the present study was to evaluate fishermen’s local ecological knowledge and conservation attitudes to help understand and collaborate in better management of the European sardine in Portugal.

## Methods

### Study area

**Fig. 1:**
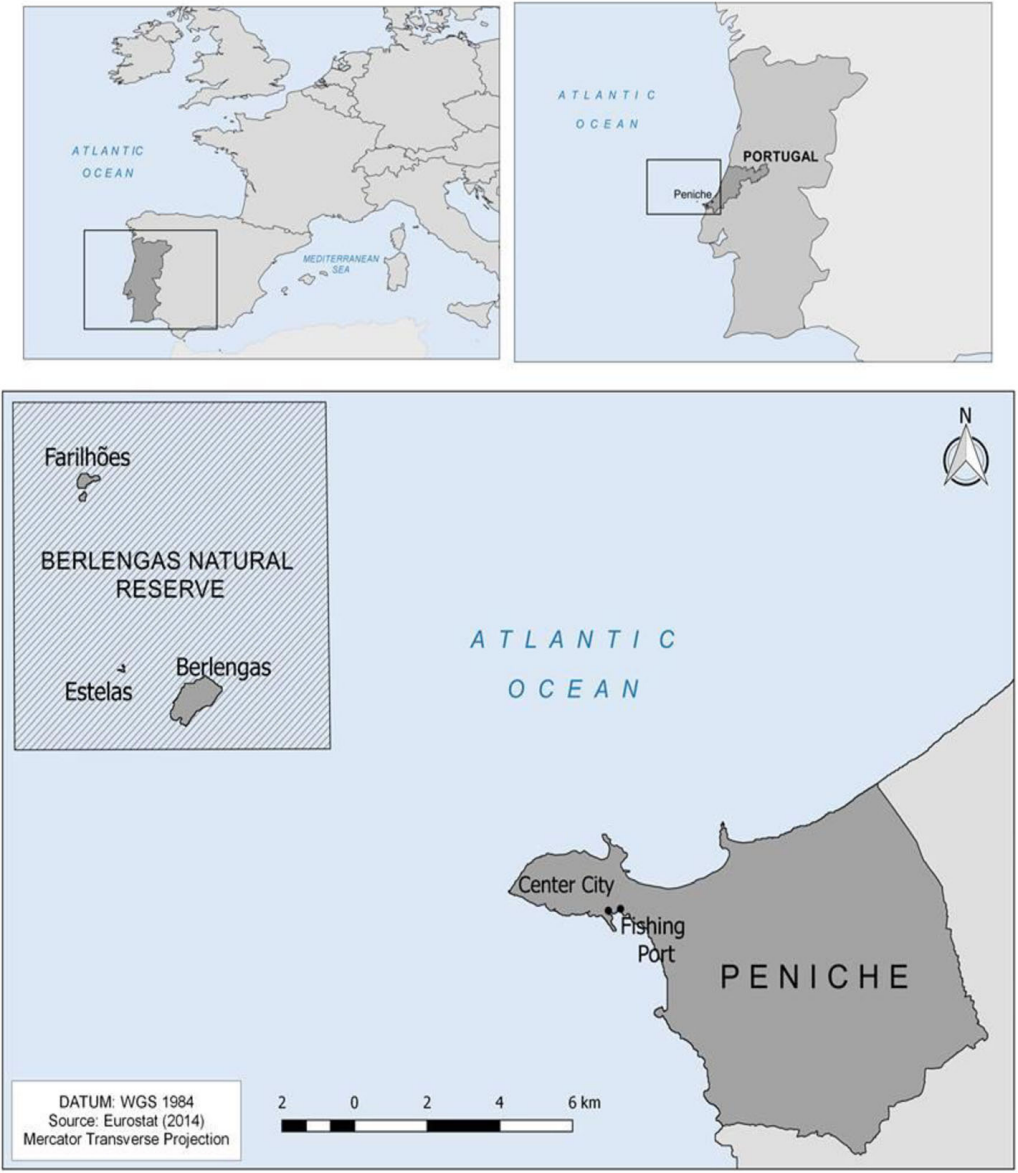
Study area of the fishery community (Peniche) on the western coast of Portugal, Iberian Peninsula. Source: Zucherato, B

This study was conducted in Peniche on the western coast of Portugal (39° 21’ 32" N, 9° 22’ 40" W; Fig. 1), approximately 10 km from the Berlenga Marine Natural Reserve (Category Ia: IUCN Strict Nature Reserve). The westernmost fishing city of the European continent [34] has an area of 77.55 km^2^ and has a resident population of 27,628 [35]. The climate of Peniche is Csb (Mediterranean), according to the Köppen-Geiger climate classification: temperate with dry, mild summers [35]. The mean annual temperature in Peniche is 16.6 °C with an annual rainfall of 873.8 mm [35, 36].

The Peniche fishing port is the most important in the central region of Portugal and the second-most important of the country when accounting for the amounts of nominal fish catches [37]. The fishing community of Peniche is one of the oldest communities with the largest number of active fishermen in the central region of Portugal [37]. Maritime authorities have registered approximately 1105 fishermen (996 in marine fishing), and fishing continues to have a significant and symbolic role in the economic and social activity of Peniche [35].

### Ethical considerations

The state-owned company Docapesca – Portos e Lotas, SA, which is responsible for the first sale of fish and supports the fishing ports sector in Portugal, granted permission for the researcher to have free access to the fishing port facilities in Peniche.

Before each interview, the respondents became aware of the objectives of the project, and the researcher requested permission to conduct the interviews. A “Statement of Informed Consent (IC)” was given to the interviewees, providing information about the researcher and the institution to which the researchers belong (see Additional file 1).

### Data collection

To evaluate the fishermen’s LEK and attitudes concerning the European sardine (*Sardina pilchardus*), individual interviews were conducted from June to September 2016 in the fishing community of Peniche. Successive visits were made to the Port of Peniche and places most frequented by fishermen in the region as a way of establishing a friendly and trustworthy ambience for interviewees. Interviews in the local community were conducted randomly, and the approach to the fishermen occurred mainly during the arrivals and departures of the fishing boats in the port of Peniche.

Data collection was done through a semi-structured interview script (Table 1) [38, 39]. Interviews were recorded through notes and eventually through the use of tape recorders [39]. The questionnaire was applied and pre-tested with fishermen from the fishing port of Aveiro, Portugal, in order to improve the criteria of validity and reliability of the interview script structure [40].

**Table 1:** Interview schedule applied in the community of Peniche, Portugal

Key points of interview	Topics
1 – Interviewee profile	Age? Level of education? Income source? Time of residence in Peniche?
2 – Fishing	Fishing experience? Fishing effort? Fishing currently?
3 – Fisherman’s LEK	Projective test (identification of the species)
	Habitat? Migration pattern? Most found depth?
	Are schooling fish?
	Age of reproduction/maturation?
	Time of spawning and breeding?
	Rapid or short growth?
	Do they have eggs, larvae in their growth phase?
	Accumulation of fat (months or season)?
	Lifetime: long or short?
	Predators? What does sardine eat?
	Time of the last biological closure?
4 – Attitudes towards conservation	Do you think it is necessary to stop fishing the sardines for a few months every year to preserve them?
	What do you do when fishing very small sardines?
	During sardine fishing, fish of various sizes can come. Should sardines of all sizes be harvested?
	Are there any natural predators of sardines in the sea?
	Can and should all kinds of fishing gear be used in sardine fishing?
	Do you catch sardines at the beginning of the year? Would you catch them this time?
	Do you fish juvenile sardines?
	Do you fish for sardines when they are breeding?
	Can any fishing net be used for sardine fishing? Is there any specific fishing net?
	What is your reaction when you accidentally catch sardines?

The four guiding points of the interview were 1) interviewee profile (age, level of education, income source, stay in Peniche); (2) fishing structure (fishing currently, fishing experience, fishing effort); (3) the fisherman’s LEK about the European pilchard (projective test, habitat, migration patterns, fish depth, schooling behavior, reproduction and spawning, growth and development, predators and prey, and fishing periods); (4) attitudes towards sardine conservation. (The fourth part of the questionnaire investigated the tendency of the respondents to take a positive, moderate or negative attitude towards the conservation of the sardine population.) Attitudes were defined in this evaluation as “a psychological tendency that is expressed by evaluating a particular entity with some degree of favor or disfavor” [41].

**Fig. 2:**
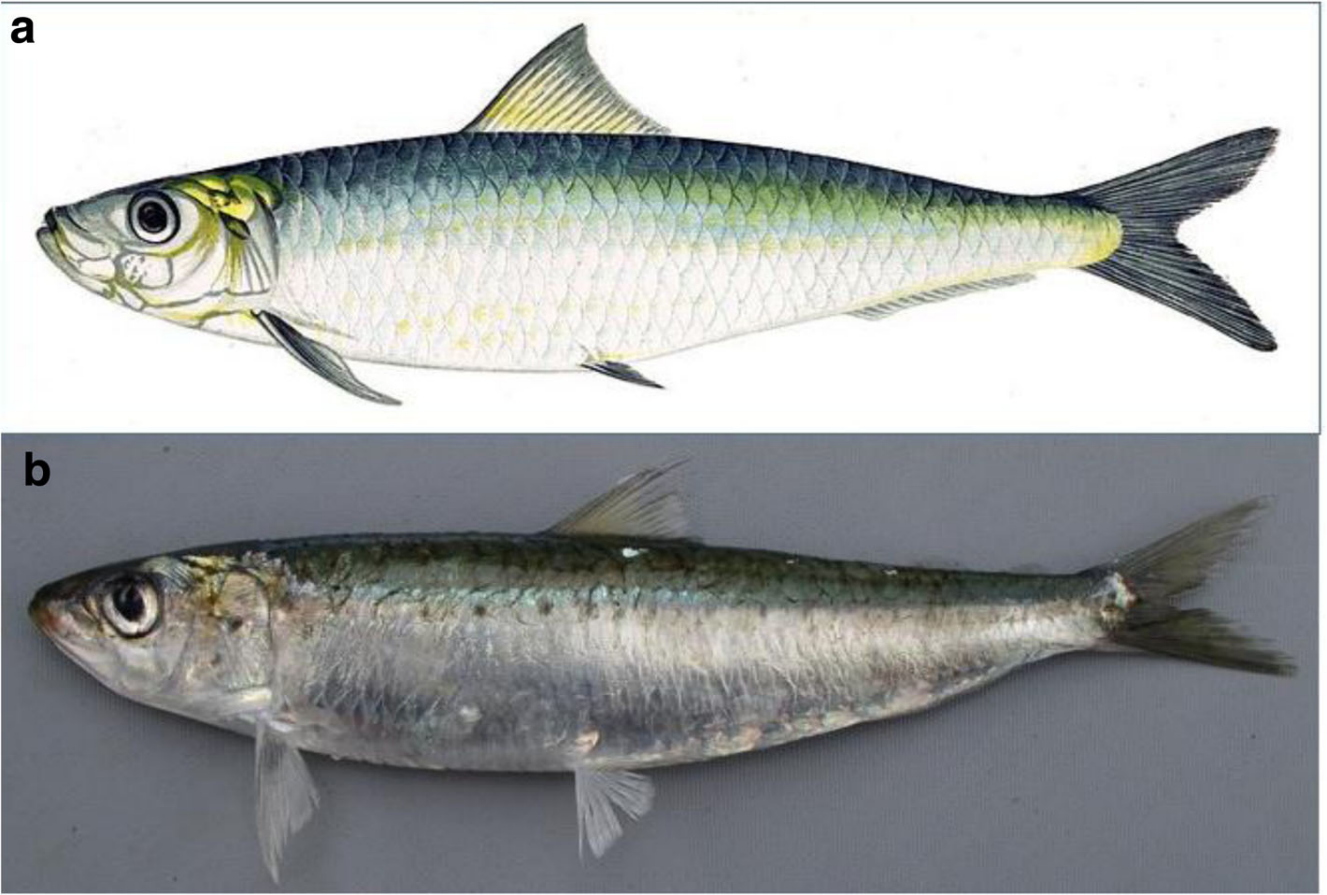
Images used in the projective test of European sardine (*Sardina pilchardus*, Walbaum, 1792). Sources: **a**: Gervais, H.; **b**: GICIM Database of the Muséum National d’Histoire Naturelle – Froese, R. and D. Pauly. Editors. 2016. FishBase

At the beginning of the third part of the questionnaire, the respondents were shown two images (a drawing and a photograph on an electronic device; Fig. 2) of the European pilchard so that they could visually identify the species and confirm the possible variations of European pilchard names. The images were presented in the same order to the respondents in this projective test [29].

### Data analyses

This ethnoichthyology research was based on an emic-etic approach between the community (local knowledge/insider view) and the researchers (scientific knowledge/outsider view) [42]. The model of integrating various individual competencies was used in the analysis of interview data, taking into account all information provided by the respondents [43]. The level of education was classified in the following classes of the Portuguese education system: primary education, A = 1st Cycle (1–4 years); B = 2nd Cycle (5–6 years); C = 3rd Cycle (7–9 years); and secondary education (10–12 years). The illiterate and higher education (more than 12 years) classes were not included in the analysis because representatives of these classes were absent from the sample.

Data on the fishermen’s LEK and attitudes towards conservation of sardines (points 3 and 4 of the questionnaire) were extracted and analyzed qualitatively and quantitatively. This analysis was performed by using a three-point Likert scale (values between 0 and 1) [44]. To analyze the levels of LEK, the correct scientific answers were scored with a value equal to 1, the answers with part of the correct answer were scored with 0.5, and the wrong answers or no response were scored with 0. For attitudes towards conservation of sardines, favorable conservation attitudes received scores with a value of 1; responses with moderate attitudes, 0.5; and responses with negative attitudes or no attitude, 0.

LEK and the attitudes of interviewees were compared quantitatively through additive indicators [45, 46]. Indicators were obtained by summing the individual score of each respondent, and this score was divided by the maximum possible score. The value obtained from each subject was calculated by afterwards by the average of all these values. The indicators of LEK and attitudes were classified in three categories (0–0.33, 0.34–0.66, and 0.67–1). LEK was classified as below average, within average or above average by comparison with the scientific literature. Attitudes were classified as negative, moderate and positive. All analyses (a one-way ANOVA parametric test, Kruskal-Wallis (H) non-parametric test and correlation analyses (r) were performed using the R Project for Statistical Computing version 3.3.2 [47].

## Results

### Fishermen’s interviews profile

Fieldwork was conducted with 87 fishermen in Peniche, Portugal, through the semi-structured questionnaire. This sample represents approximately 9% of the registered fishermen by the maritime authorities (registered activity). In this fishing community, 68% of the interviewees were born in Peniche, 17% lived in Peniche since childhood, 8% since teenagers, 6% since the age of majority, and only one fisherman does not live in Peniche, but belongs to Port of Peniche (56% in activity). Their ages ranged from 25 to 76, with a mean age of 58.3 (sd = 10.7). The years of fishing experience varied from 3 to 60, with a mean of 39.1 years (sd = 11.0). The minimum monthly income was 208 € (Euros) and the maximum 3000 €, with an average of 810.5 € (sd = 452.0 €). The average offshore fishing time was 133.83 h (sd = 498.5 h), with a minimum time of 3 h and a maximum effort of 2922 h (Table 2). The educational profile of the interviewees varied in the following classes: A (1st Cycle): 58 fishermen, B (2nd Cycle): 19 fishermen, and C (3rd Cycle and Secondary Education): 10 fishermen.

**Table 2:** Fishermen’s profile in Peniche, Portugal (*N* = 87)

	Minimum	Median	Mean	Maximum	Standard Deviation (sd)
Age (years)	25.0	60.0	58.3	76.0	10.7
Fishing experience (years)	3.0	40.0	39.0	60.0	11.0
Income source (EUR)	208.0	700.0	810.5	3000.0	452.0
Fishing effort (hours)	3.0	11.0	133.8	2922.0	498.5

### Index of LEK and attitudes towards the conservation of the European sardine

Most the fishermen (*N* = 86) correctly identified the image of the European sardine in the projective test. Only one interviewee was not sure how to identify the species. The average indicator of the LEK about European sardines for the fishermen in the fishing community of Peniche was 0.55. The median of the knowledge indicator was 0.58. The minimum indicator registered was 0.31, and the maximum was 0.77. Based on the knowledge indicator obtained, the LEK of the fishermen was classified as moderate. Only one interviewee was scored within the range of the low knowledge class, 85% were classified with moderate knowledge and 13.8% had high knowledge, when this knowledge was compared with the scientific literature.

Regarding attitudes towards the conservation of European sardines, the average indicator value was 0.76, with a minimum observed index of 0.5 and a maximum of 1. The median of the indicator was 0.78. Attitudes towards sardine conservation were classified as positive. None of the respondents registered in the range of negative attitudes, 20.7% had moderate attitudes and 79.3% had positive attitudes. Seven fishermen were scored within the maximum attitude indicator in relation to the conservation of sardines.

### LEK and attitudes: correlation and comparison analysis

Spearman’s correlation was made between the LEK and attitudes indices and between the indices and the socio-demographic variables collected in the fieldwork (ages of fishermen, fishing experience, income source and fishing time). A comparison between the indices of LEK and attitudes and the educational classes was also performed. Only significant correlations were explored in this work.

**Fig. 3:**
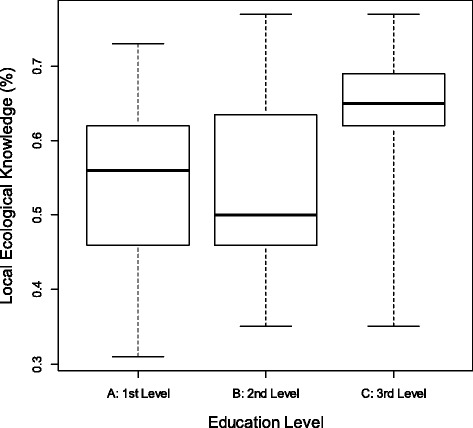
Comparison of the LEK index with the educational classes (A = 1–4 years); B = (5–6 years); C = (7–12 years); F = 3.1; *p* = 0.05; *N* = 87)

The index of LEK had a positive and significant correlation (*N* = 87, S = 79399.5, *p* = 0.0095) with the index of conservation attitudes. In this study, fishermen with a more accurate knowledge of the species tended to have more positive attitudes towards the conservation of sardines. To compare the LEK index with the differences in educational classes, the ANOVA test was performed (F = 3.1; *p* = 0.05; Fig. 3). The result showed that the differences in knowledge indices are statistically significant when comparing class A with class C but not when comparing class A with class B and class B with class C.

**Fig. 4:**
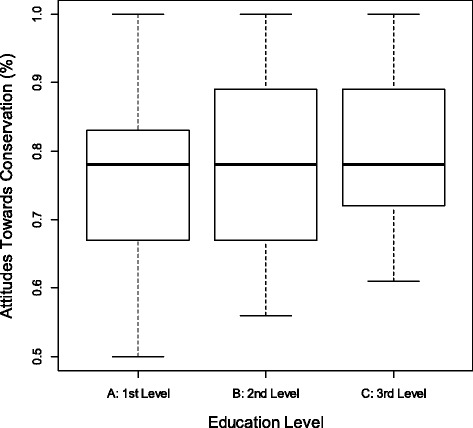
Comparison of the index of attitudes in the different classes of education (K = 124.74; *p* < 0.01; *N* = 87)

In this sample, there was a negative and significant correlation (*N* = 87, S = 137894.9, *p* = 0.016) between the age of the fishermen interviewed and the index of attitudes regarding the conservation of European sardines. It can be said that older fishermen tended to have negative attitudes towards species conservation. The Kruskal-Wallis test was performed to compare the index of attitudes in the different classes of education (*N* = 87, K = 124.74; *p* < 0.01, Fig. 4). The result showed that the differences in attitude indices are statistically significant when comparing the three educational classes. Although the medians are very close, the variation is gradually lower from class A to class C. overall, it was observed that class C is more homogeneous.

To verify in a more detailed way the behavior of the correlation of LEK index with the index of conservationist attitudes, each educational class was analyzed separately. In the first class of educational level, the results showed a positive and significant correlation (S = 23627.01, *p* = 0.037). When the two indices were compared within the second educational-level class, a positive and significant correlation was also noted (t = 2.112, df = 17, *p* = 0.04979). For the third class of education, the results showed a non-significant difference (t = -0.9558, df = 8, *p* = 0.3671).

## Discussion

### Fishermen’s LEK about the European sardine

The fishermen’s LEK in the fishing community of Peniche was moderate when compared with the scientific literature [1–10, 13, 48]. In the northern North Sea (International Council for the Exploration of the Sea – ICES Division IVa), the fishermen’s knowledge of megrim (*Lepidorhombus whiffiagonis*, Walbaum, 1792) was also potentially high and was also indicated to contribute to the evaluation and management of this species [49]. Fishermen on the Murray River, Australia, had considerable LEK about Murray crayfish (*Euastacus armatus,* von Martens, 1866) when their knowledge was compared to scientific research and fisher catch cards [50]. Ethnoecologists have observed that fishermen from Australia (North Stradbroke Island, Queensland) and Brazil (Búzios Island, Rio de Janeiro) also presented a local ecological knowledge about (*Pomatomus saltatrix,* Linnaeus, 1766) comparable to the available literature and collaborated on new hypotheses for investigation [30]. The considerable informal knowledge of pelagic fish found in the present work indicates that fishermen in Peniche seek to better understand the biology and ecology of the species, allowing them to better understand its behavior patterns and possibly to improve the quality of their fishing efforts in adverse circumstances.

The capture of sardines occurs mainly through the purse-seine fisheries along the Portuguese coast [51]. It is known that purse-seine fisheries in Portugal have sardines and other small pelagic fish as their main target [13, 48]. In the port of Peniche, the sardine plays a prominent role in the local fishing community [37]. Thus, the importance of this resource in general may have contributed to local fishermen seeking to better understand the species during the fishing effort through practical observation and dialogues with the master of the vessel. This LEK has probably been complemented by other sources of information (brochures available in the fishing warehouses, the media, and the Internet).

It is known that this ethnoichthyological knowledge of fishermen provides important information for fishery managers. Fishermen livelihoods depend directly on the marine bioresources that supports their economic, social and cultural development [28]. Traditional knowledge contributes to conservation, management and cultural sustainable practices (The Role of Culture in a Sustainable Environment) providing information about overfished species in order to a better management of natural resources [27]. Given the current uncertainties in the evaluation of the stock and the exploitation status of the sardine [18], our study supports the idea that this available and low-cost knowledge can help contribute to an adaptive management of species with scarce data sources in the integration of managers, politicians, ecologists and fishermen [52]. Moreover, a detailed analysis of the LEK available here can be used to complement pre-existing ecological data [53] as well as to understand the dynamics of the decline in fish stocks of marine species [54]. LEK data that do not correspond to the scientific literature should not necessarily be discarded. According to Drew (2005), these data can be tested as new hypotheses for new investigations.

### Attitudes towards conservation of sardines

In the fishing community of Peniche, fishermen’s attitudes about the conservation of the sardine population were predominantly positive. The same trend was observed in the Fanjingshan National Nature Reserve, southwest China, where informants had positive and supportive attitudes towards the conservation of primate species [55]. Fishermen experts belonging to two fishermen’s colonies in Ilhéus, southern Bahia State, Brazil, also had positive attitudes regarding the conservation of sea turtles [45]. Household members from the local community in the Maputo Elephant Reserve (MER) in southern Mozambique showed predominantly positive attitudes towards wildlife and conservation in that area [56].

Even though most of the fishermen in Peniche presented favorable attitudes towards conservation of the sardine population, the sardine stock in the Iberian Peninsula is still quite unstable with low recruitment levels [18]. It can be said that these positive attitudes are still recent in the fishing community of Peniche. The most negative attitudes, carried out in the past through fishing pressure along with changing environmental factors, may have contributed to the current depletion of this fishing resource.

Commonly, a system with more enforced monitoring and enforced rules has an abundance of more predictable resources, being able to economically and socially privilege the local community [57]. The imposition by the government of rules for managing a biological resource can modify the fishermen’s behavior due to the possibility of punishment [45]. However, it is known that co-management that encompasses social, economic and ecological attributes is better suited to more sustainable fishing worldwide [58]. Thus, this positive attitude towards the conservation of sardines in Peniche can also be justified by the rules imposed by the Directorate General for Natural Resources, Safety and Maritime Services of the Ministry of Agriculture and the Sea (MAS) of Portugal. The fear of possible penalties may have contributed to the presentation of more positive attitudes regarding the conservation of this biological resource in exhaustion.

Another hypothesis is that even if the fishermen of the fishing community of Peniche have a propensity to have positive attitudes towards the conservation of sardines, in practice, this attitude may not be realized. Failure to understand and manage fisheries, rather than being an important part of fisheries management, may be hindering the management of the biological resource [59].

Understanding how a local community interacts with the available biological resources in the ecosystem, based on the people’s attitudes towards and perceptions of the resource, has been increasingly used as a conservation tool [60]. The success of a management strategy should take into account the social dimension and the social acceptance of public policies and conservation strategies [61, 62]. The inclusion of this social context in the formulation of conservation measures for fish management is extremely important for its effectiveness [28]. There is an urgent need to understand how fishermen behave in regard to a biological resource and the substantial efforts that act on them [63]. This type of attitude research can help to provide a better orientation in management decisions as well as collaboration in the implementation of new policies [64].

Thus, to include fishermen from the fishing fleet of Peniche in a more active and participatory way, seeking to understand their attitudes in practice about the sardine population is indispensable at this moment. This first step may be the beginning of a new trend of participatory management in the Iberian Peninsula so that this resource can be properly managed and the approach applied in other coastal areas of Portugal.

### Knowledge, attitudes and other socio-economic variables

In the present study, there was a tendency for fishermen with more LEK about sardines to have more positive attitudes regarding conservation of the species. The knowledge of boaters in Tampa Bay in Florida has been positively correlated with attitudes supporting the conservation of the Florida manatee [65]. In Braga & Schiavetti (2013), the trend was the opposite of that found in our work. In that case, expert fishermen with more LEK about sea turtle species tended to have more negative attitudes regarding the conservation of this species [45]. In the present work, the more educated fishermen of Peniche tended to have more positive attitudes towards the conservation of sardines. Members of a rural community in a conservation area in Natal, South Africa, who were asked about conservationist attitudes about fauna and flora in this area had a propensity to have more positive attitudes with the increase of the respondent’s level of education [66]. The attitudes of the local community of Machalilla National Park in Ecuador towards the conservation and management of natural resources also had the same pattern as our study [64]. In the conservation area of Bhitarkanika, on the east coast of India, respondents with higher education showed more positive attitudes towards mangrove forest conservation, suggesting that increasing educational infrastructure is a way to improve conservationist attitudes of populations dependent on natural resources [67]. In the case of residents of the Fanjingshan National Nature Reserve in China, the educational level of the respondents had no significant effect on the attitudes [55].

The higher level of education is noted as one of the factors for the improvement of people’s attitudes in relation to conservation [64]. Gelcich and collaborators (2005) suggest that the long-term educational approach can improve fishermen’s behavioral attitudes towards a biological resource [68]. Even though the Peniche fishermen have more positive attitudes towards sardine population conservation, educational and information programs can be implemented at the Port of Peniche as a way of consolidating this attitude in the local fishing community. Economic and environmental interests should be considered in these conservation programs [69].

There is need for government to invest more in education. Programs that either fund education or technical assistance and knowledge transfer tend to improve attitudes towards conservation. The propensity for more sustainable behavior among fishermen is most likely when there is a sense between the environment and natural resources [70]. In this perspective, the local fishermen can better understand the importance of the Catch limits and closed seasons established by the European Government and thus improve their commitment to the conservation of this resource. According to Chen (2010), if fishermen are more knowledgeable about the marine environment they may be more aware and more likely to act according to the fisheries policy norms established by the imposed fishing management [70]. Thus, the introduction of monthly meetings with the participation of all involved with the natural resources in an open and participatory manner should increase positive attitudes over time [34]. At these discussion meetings, we also suggest providing information on possible changes in the behavior of species seen by both stakeholders to improve the management of this fishery resource.

Comparing the local ecological knowledge index with the different education classes, significant differences were observed only between class A and class C, although the correlation was significant (F = 3.1; *p* = 0.05). As the interviews were limited to only 3 levels of education, the pattern of relationship between the attitudes index and the educational levels was not clear. Most likely, if there was a greater variation in education, this relationship could be better explained.

We can therefore say that the local ecological knowledge about sardines in Peniche is not necessarily related to the formal education of the respondents. Because it is an active knowledge capable of responding to the presented circumstances, it can be expanded and incorporated into the generations [71], not necessarily with the increase of the education of the individual. Perhaps the second educational class of fishermen analyzed may contain individuals who have a more generalist focus on fishing. Therefore, they do not seek to better understand the behavior, ecology and biology of the species.

The older fishermen of our sample tended to have more negative attitudes regarding the conservation of the sardine population along the Portuguese Coast. The farmers’ attitudes towards the conservation of Environmentally Sensitive Areas (ESA) in the Cambrian Mountains in the southwest of England are also explained by the age of the interviewees [72]. In a local community in the District of Kuching in Malaysia, attitudes towards conservation of the deltaic mangrove forest areas were also correlated with the respondents’ ages [73]. Rural landowners in the Cape Floral Kingdom in South Africa did not show a significant relationship between age and conservation attitudes towards Overberg Coastal Renosterveld (OCR) [74].

The older fishermen in the present case may have had this negative attitude because they were more active in the past when the catches were more abundant, government restrictions and species conservation were not priorities and the income source was not committed. Once the stock reduction began to change the success of the catches [18], it became important to seek to understand the species as well as to respect its biological cycle.

## Conclusions

This is the first study in Europe regarding the evaluation of the local ecological knowledge about *Sardina pilchardus*. The fishermen of Peniche in Portugal presented moderate informal knowledge about the biology and ecology of this species when compared to the scientific literature. Attitudes towards the conservation of European sardines were predominantly positive. Fishermen belonging to the fishing fleet of Peniche with greater LEK, with a higher level of education and younger in age were more likely to bear positive attitudes involving conservation of the sardine population. The LEK is not necessarily related to the educational level of the fishermen. Nevertheless, the results showed that the differences in the knowledge indices are statistically significant when comparing class A with class C.

This study from ethnoichthyology reports comparable results with other studies that investigate the attitudes of local communities towards the conservation of natural resources [64–67] reaffirming the importance the ethnozoology and LEK as auxiliary tools in the management of natural resources [27–30].

In view of this difference, we suggest environmental education programs with the entire fishing community of Peniche to stabilize positive attitudes towards the conservation of this natural resource. Fishermen with less educational instruction and older fishermen should have priority in the suggested conservation program. Dialogue between stakeholders (local actors, managers of local fishermen’s associations, scientists and lawmakers), without privileging any parties, is advised for a better conservation strategy [72, 75].

Due to the large gap in the scientific literature on LEK in the Iberian Peninsula, and especially in Portugal [76], we suggest new studies of the same scientific nature in the Iberian Coast as a way of comparing our findings. The local ecological knowledge about European sardines that was considered incompatible with the scientific literature should not be completely discarded. This LEK can be used to formulate new hypotheses for future scientific research [32, 77]. Such research, if well conducted, can help in better understanding the natural resource in question and assist in the recovery, management, action, and adjustment of the European sardine fishery on the Portuguese Coast.

## Additional file

Additional file 1: Statement of Informed Consent (IC) and agreement to participate in the research. (DOCX 17 kb)

## Abbreviations

ESA: Environmentally sensitive areas
IC: Informed consent
ICES: International council for the exploration of the sea
LEK: Local ecological knowledge
MAS: Ministry of agriculture and the sea
MER: Maputo elephant reserve
OCR: Overberg coastal renosterveld
